# Tissue engineering of collagen scaffolds crosslinked with plant based polysaccharides

**DOI:** 10.1007/s40204-021-00149-4

**Published:** 2021-02-18

**Authors:** Rohit Rekulapally, K. Udayachandrika, Sirisha Hamlipur, Anuja Sasidharan Nair, Biswajit Pal, Shashi Singh

**Affiliations:** CSIR Centre for Cellular and Molecular Biology, Uppal Road, Hyderabad, 500007 India

**Keywords:** Extracellular matrix, Cross-linked collagen, Polysaccharides, Biocompatibility, Cytotoxicity, Differentiation

## Abstract

Ideally, a bioscaffold should mimic the characteristics of an extracellular matrix of a living organ of interest. The present study deals with the formation of composite scaffolds of collagen with gum arabic. Collagen was cross-linked with oxidized gum arabic having aldehyde groups to form a porous block. By changing the oxidation level of gum arabic, incorporation of the polysaccharides into the scaffold could be varied resulting in scaffolds with variable polysaccharide to protein content. A series of scaffolds were made by altering collagen concentration and oxidation level of gum arabic. The scaffolds were tested for their physical properties, stability, biocompatibility and ability to support the cell growth. Results implied that variable polysaccharide incorporation into the scaffolds was possible depending on the oxidation level of gum arabic which could influence the swelling behavior. The scaffolds showed non-toxic behavior towards the mesenchymal stem cells and nucleus pulposa cells using viability assay in culture conditions up to 30 days; the growth of cells was seen at all combinations of gels. Nucleus pulposa cells were able to maintain their phenotype in the GACO gels. The studies show that these scaffolds are potential candidates in applications, such as tissue engineering, and can be designed to match the requirement of different cell/tissues as per their ECM.

## Introduction

Tissue engineering involves creating organoids using cells in a 3D milieu made up of scaffolds and other important ingredients like growth factors. It is ensured that the materials in construct are not just inert fillers but rather dictate cell function and character transformation to the desired phenotype. The cells and the scaffolds/substrates have reciprocal influence over each other resulting in homeostasis over a period of time. Field of tissue engineering/regenerative medicine is replete with numerous scaffolds designed from a number of biomaterials and equally large variety of fabrication strategies. Host biocompatibility and bioactivity of natural polymers or extracellular matrix (ECM) components give these an edge over other materials. Extracellular matrix is a product of locally secreted proteins and polysaccharides arranged in a cell/tissue-specific combination, pattern/topology and mechanical properties. Among these, polysaccharides and proteins have been frequently used with minimal or no toxicity, good cell interaction and appropriate physical properties (Ungaro et al. 2005; Lapidot et al. 2012).

A wide variety of polysaccharides available from natural resources can be used in combination with natural and synthetic biopolymers or inorganic material for adhesion, growth or differentiation of cells (Mano et al. 2007; Bacakova et al. 2014). Not all polysaccharides have been found suitable but starch, cellulose, chitin, chitosan, guar gum, alginate, agar, glycose-amineglycans, carboxy methylcellulose are some of the polysaccharides reported in designing scaffolds for various cell types (Camponeschi et al. 2015; Tiwari et al. 2019; Silva et al. 2015; Edgar et al. 2016).

Gum arabic (GA) derived as plant exudates from acacia plant, consists of a variable mixture of arabinogalactan, oligosaccharides, polysaccharides and glycoproteins forming a physiologically harmless substance. The chemical composition of GA is complex and consists of about 97% carbohydrates. Idris et al. (1998) reported GA to be composed of 39–42% galactose, 24–27% arabinose, 12–16% rhamnose, 15–16% glucuronic acid, 0.22–0.39% nitrogen, and 12.5–16.0% moisture) and about 3% proteins. GA has not been reported much for its use in tissue engineering but exhibits emulsifying, encapsulating and film forming abilities that help in drug delivery and giving stability to nanoparticles (Ali et al. 2009). GA provides functional groups for coupling, thus helps in functionalization of nanomaterials (Palma et al. 2015). Owing to the presence of sugars, gum arabic can also be oxidized, creating reactive groups for cross-linking with other polymers/proteins, etc. (Ehrenfreund-Kleinman et al. 2002; Nishi et al. 2005; Sarika et al. 2014, 2016). We have used an oxidized form of gum arabic to enable cross-linking of collagen scaffolds.

Among the extracellular matrix proteins, collagen is the major protein providing structural support, strength and stability to all organs depending on the functional requirement of each tissue. Collagen along with other polymers/proteoglycans/polysaccharides/inorganic salts like hydroxyapatite has been used in various tissue constructs (Mano et al. 2007, O’Brien et al. 2011; Glowacki and Mizuno 2007). Collagen can be used as gels, as such or in cross-linked form, either way it is found to be biocompatible and non-immunogenic. Collagens naturally support cell adherence, but have less mechanical strength that can be improved with cross-linking. Besides, collagens are liable to rapid degradation. Most of the cross-linking agents have known toxicity and their use in in vivo applications remain limited. Cross-linked collagen in combination with other biomaterials has been quite popular for designing scaffolds. Composites are advantageous in terms of physical properties and biological outcomes due to synergistic effect of individual components (Rodrigues et al. 2012).

ECM of each organ/tissue is specific and dictates the character and function of the cells. The main constituents of the ECM are proteins, glycoprotein and glycans. Carbohydrates make an essential component of the ECM influencing its structure, mechanical properties and function. In the present work, we have used natural polymers, namely collagen and gum arabic (GA), to create a series of scaffolds altering their protein to polysaccharides ratios and crosslinks. GA in aldehyde form with variable level of oxidation, was reacted with collagen to prepare a series of cross-linked hydrogel. These gels were tested for stability and biocompatibility. We present a novel concept to customize tissue-specific scaffolds by altering the oxidation level of polysaccharide or concentration of collagen. The purpose of the study was to demonstrate that by changing the oxidation level of crosslinking polysaccharide and/or concentration of collagen, a variety of scaffolds can be produced that may suit the requirement of different tissues. The scaffolds display differential response to growth of the cells.

## Materials and methods

All the materials, chemicals, etc. used in the paper were procured from the sources mentioned. Gum arabic, sodium periodate were procured from Sigma Aldrich. 2,4-dinitrophenyl hydrazine (DNPH) and para-dimethylaminobenzaldehyde (pDMAB) from Merck Millipore. Collagen was routinely extracted in the laboratory from rat tails and checked for purity by SDS-PAGE. MTT reagent was purchased from Invitrogen.

### Preparation of gum arabic aldehyde

Gum arabic aldehyde (GAA) was prepared from gum arabic (GA) [Sigma Aldrich] using sodium periodate [Sigma Aldrich]. A 10 g of arabic gum was dissolved in 70 mL of water and 0.86 g to 5.16 g of sodium periodate in 30 mL solution was added making it up to 100 mL. The amount of periodate was varied to acquire different degrees of oxidation of GA. The reaction mixture was stirred continuously under dark at RT for 6 h and purified by precipitation using acetone. The precipitate was frozen and lyophilized. The lyophilized product was weighed and the yield was estimated based on percent ratio of oxidized product to GA used.

1 $${\text{Yield}} = {\text{weight of gum arabic aldehyde}}/{\text{weight of gum arabic }} \times 100.$$

The yield of the product was in the range of 80–120%. The lyophilized powder was dissolved in 0.1 M borax solution to make a 10% solution of GAA (*N* = 20).

For estimation of aldehyde, we used 2,4-dinitrophenylhydrazine (DNPH) [Merck-Millipore] (Tummalapalli et al. 2015). 5 µL of each standard (formaldehyde solution (10–40%) and samples were added to 500 µL of DNPH solution. Reaction mixture was centrifuged at 12,000 rpm after 1 h and the supernatant was measured for transmission at 357 λ using Multimode spectrum (Thermo Electron corporation) plate reader. The amount of aldehyde in our samples was calculated after subtracting from the total dye added and converted to moles/g (*N* = 20).

### Preparation of collagen/gum arabic aldehyde scaffolds

To prepare the scaffold, 5 mL of solution of 10% oxidized gum arabic was mixed with 5 mL of (1–4%) collagen solution and vortexed for 30 s. The above solution turned into a thick homogenous emulsion that was added into dishes and allowed to solidify. Time taken for the gel to solidify was also observed.

### Characterization of GA-CO scaffolds

Scanning electron microscopy was used to visualize morphology of the gum arabic-collagen (GACO) scaffolds. Lyophilized scaffold was coated with gold in vacuum sputter coater and analyzed in Hitachi SEM (Japan).

FTIR analysis was done to confirm the gum arabic aldehyde (GAA) formation and interaction of GAA and collagen in scaffold. The sample was placed on the attenuated total reflection (ATR) crystal (ZnSe ATR crystal) of FTIR spectrometer (Bruker Optics) and a transmission spectrum ranging from 400 cm^−1^ to 4000 cm^−1^ was taken for 200 scans keeping a resolution of 4 cm^−1^. The spectra obtained are analyzed by Opus software/IgorPro.

Porosity of the scaffolds was measured according to method of Li et al. (2008). Dry scaffolds were weighed and then immersed in ethanol for 2 h. After 2 h, the weight of each scaffold was measured while keeping them suspended in ethanol and further weighed after removing from ethanol. The porosity was calculated using the following formula:

2 $${\text{Porosity }} = W_{2} - W_{3} - Ws/W_{1} - W_{3} ,$$

where *W*_s_ is the weight of dried sample, *W*_1_ is the weight of alcohol, *W*_2_ is the weight while suspended in ethanol, *W*_3_ is the weight of liquid after removing the block.

Stability of the formed scaffolds was checked by the degradation profile of scaffold blocks left in sterile PBS for up to 4 weeks. Weighed samples of about 1 g (av 1.12 + 0.15 g) each were immersed in 10 mL PBS in a tight container and left at RT. After 4 weeks, the samples were lyophilized and weighed again. Weight ratio was calculated for each set of block (*N* = 4).

Degradation of the scaffolds was measured after leaving the scaffolds of about 100 mg (av 116.8 + 0.038 mg) each in protease solution (4 mg/10 mL) for 24 h, followed by lyophilization and weighing. The protease treatment was repeated three times and overtime the degradation rate was calculated by the weight ratio percentage.

### Estimation of carbohydrate content and collagen

The amounts of carbohydrate and collagen contents of the scaffolds were estimated. With a known quantity of scaffold, the carbohydrates were measured by method of Varkhade and Pawar (2013). Briefly, the blank, the standards (glucose 60–100 µg/mL) and the samples (~ 1 µg/100 µL) were mixed with 100 µL of 5% phenol and 500 µL of conc. sulphuric acid. After 10 min, OD was taken for each tube at 488 nm in the UV visible multi-mode plate reader (Thermo Electron Corporation). The amount of carbohydrate in sample was calculated from standard calibration curve and expressed as amount per gram of scaffold.

Collagen/protein estimation in scaffolds was carried out with *para*-dimethylaminobenzaldehyde (*p*DMAB) after alkali hydrolysis. Briefly, the blank, pure collagen as standard (50–100 µg) [Sigma–Aldrich] and a known amount of scaffold of ~ 10 mg in each case was used for estimation. After alkali hydrolysis with 100 µL of 10 N NaOH at 120 °C, the sample was neutralized with HCl. 100 µL aliquot was taken and dried in multiwell plates and treated with chloramines T at RT. After incubation with 100 µL of pDMAB 60 °C for 25 min, readings were taken in the UV–visible multi-mode plate reader (Thermo Electron Corporation) at 550 nm. The amount of protein was calculated from calibration curve and expressed as amount/g of scaffold.

### Cell culture studies

Mesenchymal stem cells derived from human placenta (Thejaswi et al. 2012) and IVD (Intervertebral disc) cells obtained from human spine disc (nucleus pulpous) samples from patients undergoing surgery were used in the study. All the samples were collected with informed consent of the patient after obtaining IRB approval from the hospital; Ethical committee and Institutional committee for Stem Cell research of our institute.

Scaffolds were sterilized by rinsing with ethanol and placing them in biosafety hood with the UV for 1 h. Before seeding the cells, scaffolds were rinsed with PBS and culture medium. Cells were plated directly on the scaffolds and cultured in DMEM with 10% serum. The dish was kept in CO_2_ incubator at 37 °C with 5% CO_2_. Cells were examined in Leica Confocal microscope after staining with Hoechst 33342 on day 2, 10 and 21. Then, the cells were imaged at different heights. After 15 and 30 days in culture, some of the tissue constructs were used for histology and immunohistology. Analysis was performed using LEICA Application suite X version 2.0. 2.15022.

Scaffolds with cells were also processed for SEM imaging. Scaffolds were fixed in 2% glutaraldehyde, followed by dehydration, critical point drying. Dried samples were mounted and gold-coated before examining in SEM.

Viability of mesenchymal stem cells was examined by MTT assay. The cells were seeded on a scaffold at 30,000 cells/cm^3^ in DMEM containing 10% FBS and kept at 37 °C in a 5% CO_2_ atmosphere for 24 h. Cells cultured without any scaffold were used as control. After 24 h, 800 µL of MTT solution (5 mg/mL) was added into each sample and incubated for 4 h. After 4 h, 0.01 N HCl dissolved in isopropanol was added and incubated for 40 min. The plate was centrifuged and supernatant was transferred to fresh dish and the color product developed was quantified by measuring absorbance at 570 nm using spectrophotometer.

## Results and discussion

### Oxidation of gum arabic

**Fig. 1:**
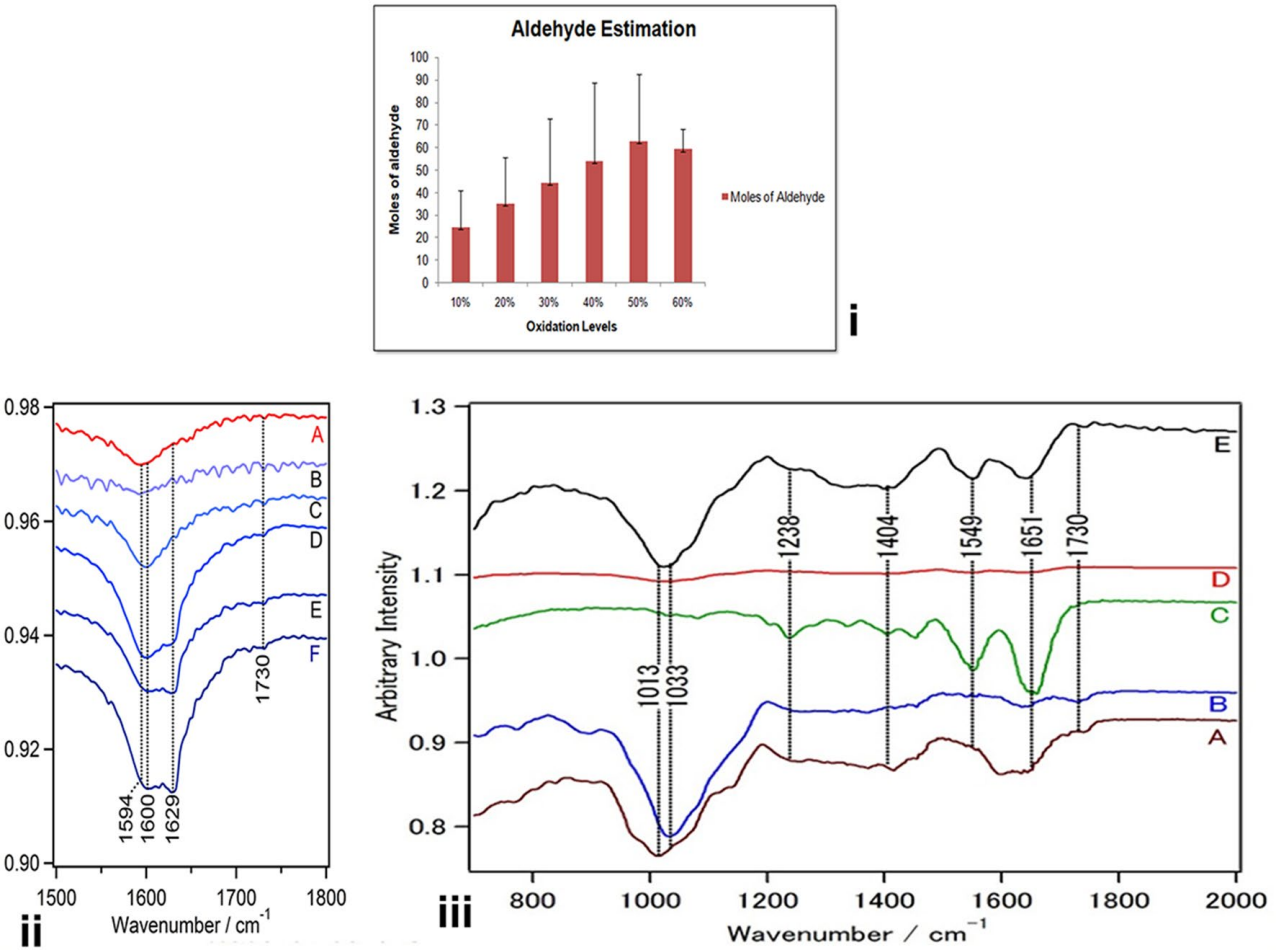
(i) Aldehyde levels in oxidized gum arabic. The gum arabic oxidized for 50 and 60% show maximum aldehdye content. FTIR of oxidized gum arabic at different oxidation levels (ii) and (iii) spectroscopic analysis of the composite scaffold and its raw components. **a** IR spectra of gum Arabic. **b** Aldehyde form of gum arabic shows appearance of a band at 1730 cm^−1^assigned to aldehyde stretching. **c** IR spectra of Collagen showing a helical structure with characteristic amide bands at 1651 cm^−1^, 1549 cm^−1^ and 1238 cm^−1^. **d** Shows the structure of scaffold and its amplified form (10 ×) in **e**

**Fig. 2:**
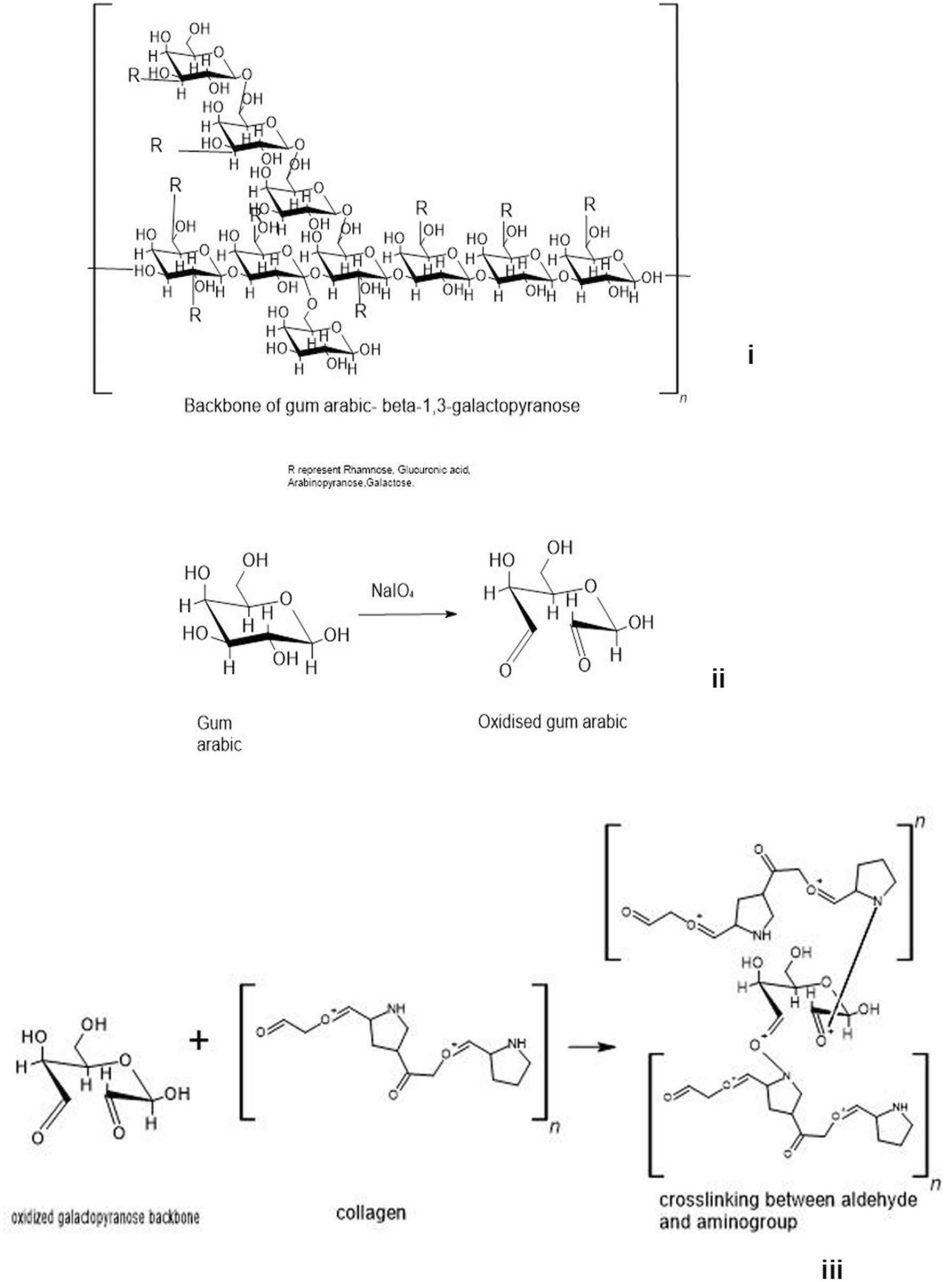
Scheme of the structure of gum arabic (i), oxidation of sugar component of gum arabic (ii), and linkage of CHO- group with collagen molecules (iii)

Gum arabic (GA) was oxidized with periodic acid to generate aldehyde groups on this complex polysaccharide. GA was oxidized with variable amounts of periodic acid to generate different levels of oxidation (10–60%) as given in Table 1; GA (40 mmol) and periodate were mixed in molar ratios so as to acquire a 10, 20, 30, 40, 50 and 60% oxidation. The presence of aldehyde was confirmed in a rapid test with Schiff’s reagent that turned the solution pink. The gum arabic aldehyde (GAA) was lyophilized and made up to a 10% solution in borax buffer. Quantitative analysis of the aldehyde content in GAA showed the presence of aldehyde groups at all levels of oxidation. The moles of aldehyde per gram of oxidized group increased with an increase in the amount of periodate added to reaction indicating increased level of oxidation (Fig. 1i). Yield of the product also increased with increased level of oxidation (Table 1). GAA prepared with 50–60% oxidation had maximum amount of mol/g of aldehyde. Gum arabic is polysaccharide-rich plant exudate containing polysaccharides and proteins. Owing to the presence of sugar moieties, it is oxidized with periodate to generate the aldehyde groups (Fig. 2i,ii), and these can be conjugated to imine/amine groups of other biopolymers by Schiff’s reaction (Fig. 2).

**Table 1:** Amount of periodate used for oxidation and yield of oxidized product

Oxidation level (%)	Amount of periodate per 100 mL of reaction	Percent yield of product
10	0.86 g (4 mmol)	75 ± 3.6
20	1.72 g (8 mmol)	85 ± 5.8
30	2.58 g (12 mmol)	94 ± 8.2
40	3.44 g (16 mmol)	116 ± 6.1
50	4.3 g (20 mmol)	122 ± 8
60	5.16 g (24 mmol)	123.5 ± 12.6

### Preparation of scaffolds

A 10% solution of oxidized gum arabic (at various oxidation levels of 10–60%) dissolved in borax buffer was mixed with 1–4% collagen solution in ratio of 1:1 and mixed thoroughly on vortex till its frothy mixture turned viscous which before setting, it was poured into multi-well dishes. The blocks once set, were washed free of soluble impurities (contaminants) and lyophilized. It was observed that there was a time difference in setting of gels as the oxidation level of GAA increased and also with increase in concentration of collagen. The gel with 4% collagen cross-linked with a 60% oxidized GAA was difficult to pour as it would set by the time within 1 min, whereas the gel with 1% collagen with GAA of 10% oxidation level would take about 15 min to completely solidify (Table 2). The longer gelation time with low oxidation level of GAA, the low collagen concentration may be due to less cross-linking. At higher oxidation levels of GAA, there will be higher availability of functional groups for cross-linking.

**Table 2:** Time of gelation after mixing the two components in equal proportion

Concentration of collagen (%)	Gelation time in minutes					
	Oxidation level of gum arabic					
	10%	20%	30%	40%	50%	60%
1	10.47 + 0.19	10.0 ± 1	6.40 ± 0.13	5.20 ± 20	3.00 ± 23	2.03 ± 0.11
2	4.15 ± 0.21	2.53 ± 0.6	2.34 ± 0.2	2.05 ± 0.2	1.54 ± .20	1.45 ± .20
3	2.55 ± .56	2.17 ± 0.16	1.56 ± 0.11	1.50 ± .45	1.42 ± .35	1.05 ± 15
4	1.45 ± 0.33	1.38 ± 0.40	1.22 ± 0.05	1.02 ± 0	0.59 ± .07	0.49 ± .08

Oxidized gum arabic dissolved in borax buffer was mixed with 1–4% collagen solution in ratio of 1:1 and vortexed for about 30 s when the frothy mixture turned viscous and was poured into multi-well dishes before they set. Schiff’s base reaction involving the aldehyde and amine group is a favorable strategy for cross-linking in mild reaction conditions (Fig. 2). Borax buffer would also stabilize the oxidized GA by reducing the unstable imine bonds. The reaction carried out in the presence of borax has an additional advantage by forming a complex with polysaccharides/polymers through ligands containing diol or triol groups. These intermolecular complexes work as cross-links (Shao et al. 2000). The dual cross-linking reaction was used to cross-link collagen creating 3D scaffolds discs.

### FTIR of scaffold formation

To characterize the oxidized gum arabic, IR spectra were collected in the range of 750–2000 cm^1^ and presented in Fig. 1. Normally, C–O stretching (ether) in the IR spectra appears between 1000 and 1300 cm^−1^ and the band at 1016 cm^−1^ is assigned to C–O stretching (C–O–C). The presence of 1594 cm^−1^ band indicates carboxylate symmetric stretch, whereas the 1412 cm^−1^ band indicates asymmetric stretch as seen in spectra of gum arabic by Sarika and James (2015) and Ibekwe et al. (2017). Infrared spectrum analysis of the oxidized gums revealed a slight increase in the intensity of the peak in the region of 1725–1735 that identifies aliphatic aldehydes, which were not identifiable in the normal or unoxidized gum arabic. In similar studies by Sarika and James (2015) and Ali et al. (2018), the aldehyde peak appeared around 1738 and 1728. Apart from this, there was a slight shift in the peak intensity at 1594–1629 in the oxidized GA with increasing the oxidation levels (Fig. 1ii–iiia, b), which would be due to symmetric stretching of COOH (carboxylic group) of uronic acid residues, because of aldehyde formation also seen in FTIR spectra of oxidized gum arabic by Ali et al. (2018).

Collagen has a helical structure and is also clearly identified in the IR spectrum (Fig. 1iii, c), 1651 cm^−1^ being amide I and 1549 cm^−1^ as amide II, whereas 1238 cm^−1^ indicates amide III. Spectrum of the scaffold is shown in Fig. 1iii, d and has been enhanced 10 times in Fig. 1iii-e. In this spectrum, the amide I and amide II bands are clearly visible along with the presence of C–O stretching (C–O–C), which appears between 1013 cm^−1^ and 1033 cm^−1^, indicating the formation of the scaffold. Disappearance of 1725–35 cm^−1^ band in the scaffold indicates that the aldehyde group is involved in the scaffold formation (Fig. 1iii b, e). Similar changes were also reported by Sarika and James (2015).

### Properties of scaffold

**Fig. 3:**
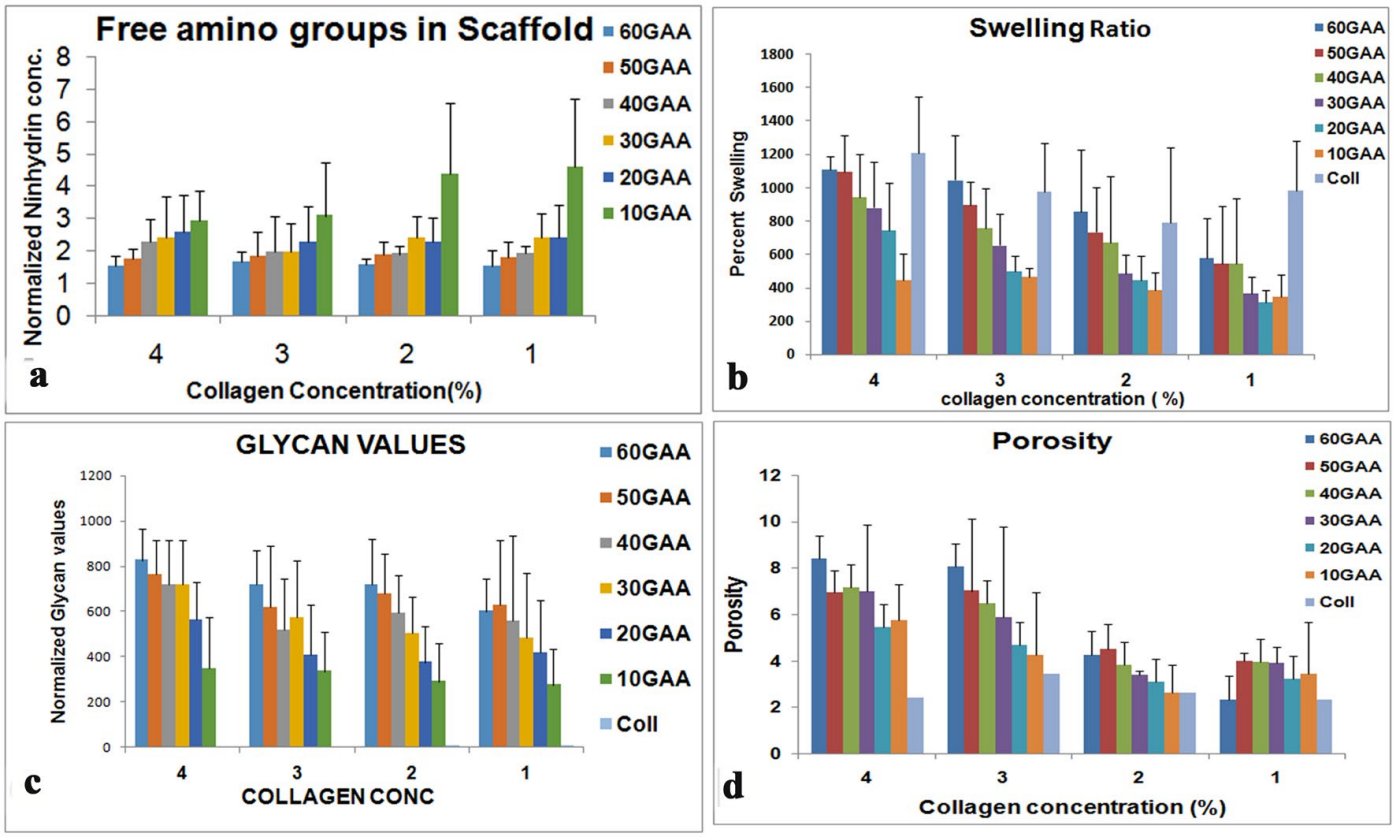
Properties of the scaffold. **a** Amount of free amino acid available in cross-linked scaffold. The free amino acid declines with increasing oxidation level of gum arabic. **b** Swelling ratio of the scaffolds-collagen shows high swelling capacity as compared to crosslinked scaffold but in the scaffold the swelling ratio increases with high oxidation level of gum arabic. **c** With increasing oxidation level of gum arabic the amount of polysaccharide incorporated in the gel increases. **d** Porosity of the gel also shows an increase with increasing oxidation level of gum arabic

With more aldehydes in the polymer, it was expected to have more capacity to link to amine groups of the proteins. Degree of cross-linking collagen with gum arabic was assessed by measuring the free amino groups in the gel by ninhydrin assay. The amount of free amino acids declined significantly (*p *− 0.0001) with increase in oxidation level at all concentrations of collagen (Fig. 3a). There was not much significant change in values of free amino groups with collagen concentration (*p* 0.7). As more aldehyde groups are available in GA with higher oxidation level (Fig. 1i), the amine groups are utilized in the gels with higher level of oxidized GAA.

Swelling is an attribute that depends on the extent of cross-linking, a well cross-linked gel is supposed to have less swelling capacity. Swelling of tissue engineering scaffold is a crucial property as it would influence the pore size, hydration of the scaffold material. Swelling influences diffusion of medium containing nutrients, oxygen and other metabolites. The swelling property of the gels, prepared by different levels of GA oxidation and collagen concentration, was examined. The GACO scaffolds were left in PBS for 24 h and up to 48 h; the weight of the scaffold was estimated. All the scaffolds attained equilibrium by 24 h. Collagen blocks without GAA had higher swelling capacity as compared to the scaffolds cross-linked with GAA (Fig. 3b). Among the GACO gels, the swelling ability of the GACO gels was higher in gels with higher GA oxidation and higher collagen concentration. The graph showed at each concentration of collagen the swelling ratio increased with higher oxidation level (*p* 0.0005). Though this was contrary to our expectation, we attributed this property of water holding to enhanced incorporation of the polysaccharides in the gel. Presence of polysaccharides enhanced the hydration capacity (Yanagishita 1993). The gels at all combinations were examined for the polysaccharide content in the lyophilized gels.

The amount of polysaccharide, incorporated into the Col-GA blocks (scaffold), increased with increasing oxidation levels making it more hydrophilic for each concentration of collagen (*p* < 0.0001). The polysaccharide levels differed across different collagen concentrations, reducing gradually from 4 to 1% (*p* = 0.0086); (Fig. 3c).

Collagen cross-linking is generally carried out using bifunctional cross-linkers like glutaraldehyde or carbadiimide that are toxic to cells and not favorable for in vivo use (Osborne et al. 1998; Davidenko et al. 2015). Using GAA for cross-linking also supplements the scaffolds with additional polysaccharide groups that are routinely abound in extracellular matrix (Mano et al. 2007; Frantz et al. 2010). Hydrophilic nature of scaffolds is of immense importance as it would determine the level of media adherent to the scaffolds at the time when cells are seeded on scaffolds. Glycans are responsible for the mechanical stability, capacity to retain water and resistance to compressive forces in many tissues. This mode of cross-linking helps in making stable scaffolds. GACO scaffolds were quite stable up to a month in PBS or culture medium.

### Morphology and porosity of scaffold

**Fig. 4:**
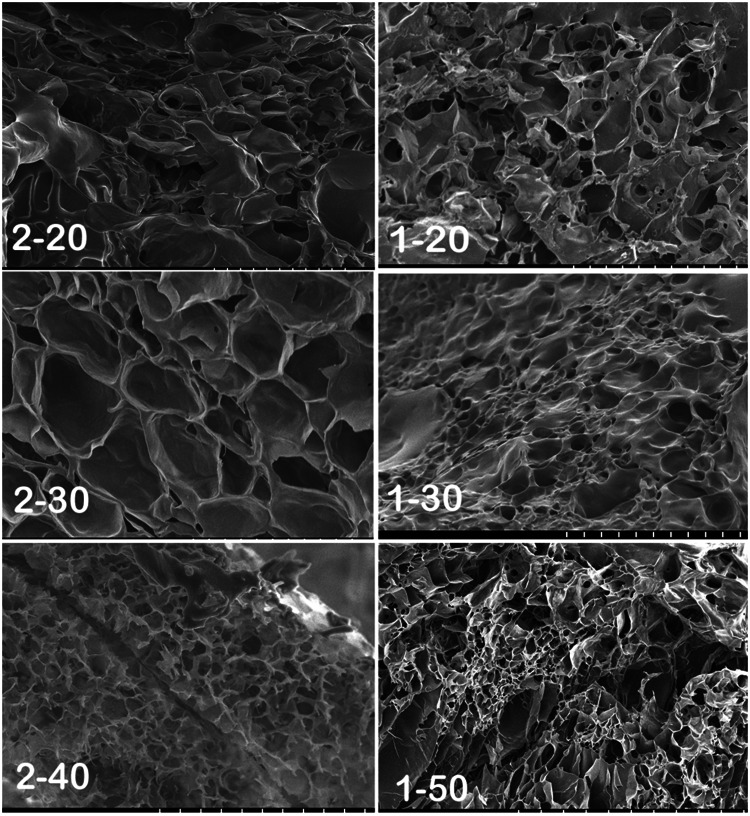
Scanning electron microscopy of collagen gels cross-linked with oxidized gum arabic. All the gels were porous with variable pore size gels with higher oxidation level tended to have smaller pores. Left panel has gels with 2% collagen with gum arabic (GA) oxidized up to 20, 30 and 40%; gels on right are with 1% collagen with GA at 20, 30 and 50% oxidation level

An ideal scaffold is supposed to be porous to the extent that the cells can thrive inside it and can differentiate under appropriate conditions. Morphology of a collagen block was totally different from the GACO scaffold. Scaffold blocks at all combinations were found to be highly porous upon examination in scanning electron microscope. Scaffolds exhibited porous morphology over several layers resulting in a connected honeycomb-like structure in all combination of gels. Though the pores were variable in size (Fig. 4), the size of pores appeared to depend upon the combination of collagen concentration and GAA oxidation level. The pores in gels with higher oxidation of gum arabic (Fig. 4; 2–40; 1–50) were relatively smaller in appearance compared to pores in Fig. 4; 1–20. Porosity of the scaffolds was estimated by liquid displacement method using ethanol. In this study, with increasing oxidation level in scaffolds, the porosity goes on increasing. Scaffolds with 60% oxidation showed maximum porosity at all collagen concentrations (*p* = 0.0005); (Fig. 3d). Increase in collagen concentration also resulted in more porosity (*p* = 0.03463).

### Stability of the scaffolds

Degradation studies hold a paramount importance while preparing a potential scaffold for tissue engineering purposes. Level of degradation is ultimately going to determine the stability of scaffold at the time it will be harboring cells along with the culture media. This also plays a role during in vivo healing, as the scaffold should be stable for ample time for the body to recover the functionality of the implanted structure; and also by then, the cells are able to secrete their own ECM.

**Fig. 5:**
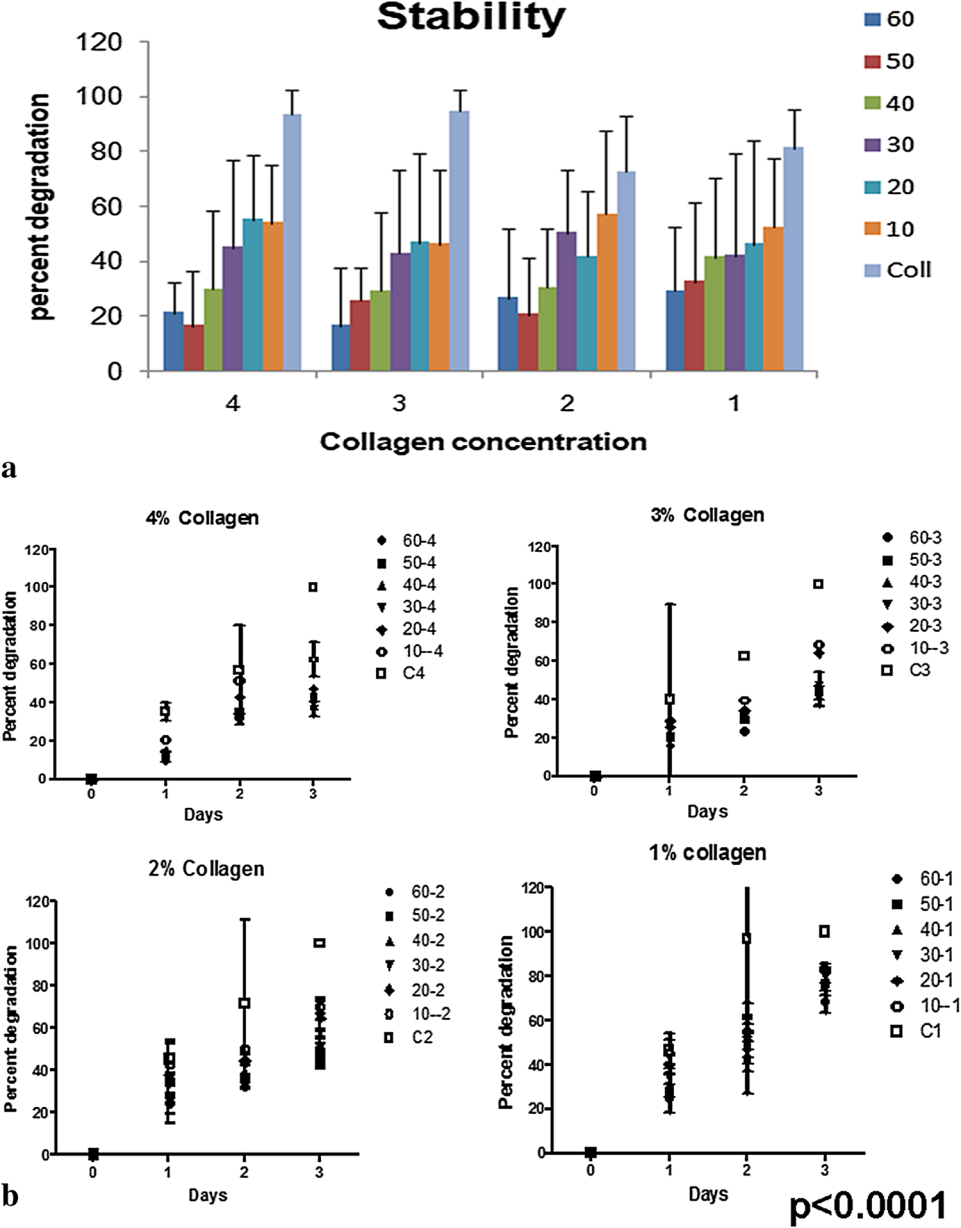
Stability of the gel (i) The gels left in sterile PBS for over 4 weeks showed least degradation in gels with higher oxidation level and high concentration of collagen. The degradation was faster in uncross-linked collagen. (ii) Parallel studies of gels exposed to protease also showed a similar trend of faster degradation of uncross-linked collagen. Scaffold with low oxidation level of gum arabic were faster to degrade

Stability studies were done using weight methods and it was concluded that the scaffold prepared at 50–60% oxidation level remained stable with least amount of degradation. Stability studies were carried out with about 1 g of scaffold until the non-crosslinked collagen blocks had degraded, i.e., until day 25. At each concentration of collagen, the stability increased with increased oxidation. The stability can be attributed to high level of cross-linking which increases with the increase in oxidation level of polysaccharide, as the plain collagen with no cross-linking polysaccharide degraded the maximum, till it could not be assessed (Fig. 5).

Degradation properties of the scaffolds were examined in parallel by incubating the gels (about 100 mg) in protease solution and checking its solubility. This was important as the scaffold if implanted would be exposed to the biological fluids and proteases. This would mimic the in situ milieu. The graph shows that the collagen scaffolds without GAA degraded by 35% in day one itself and were completely degraded by day 3 (Fig. 5). The cross-linked gels demonstrated more stability and less degradation depending on concentration of GAA in the gel.

Results indicated that the scaffolds prepared at 60% oxidation level showed least degradation and this can be of great importance while designing a potential scaffold exhibiting great amount of stability (Fig. 5). In tissue engineering, there is always a need to design scaffolds which will remain stable over the entire period proper function.

### Cell culture studies

**Fig. 6:**
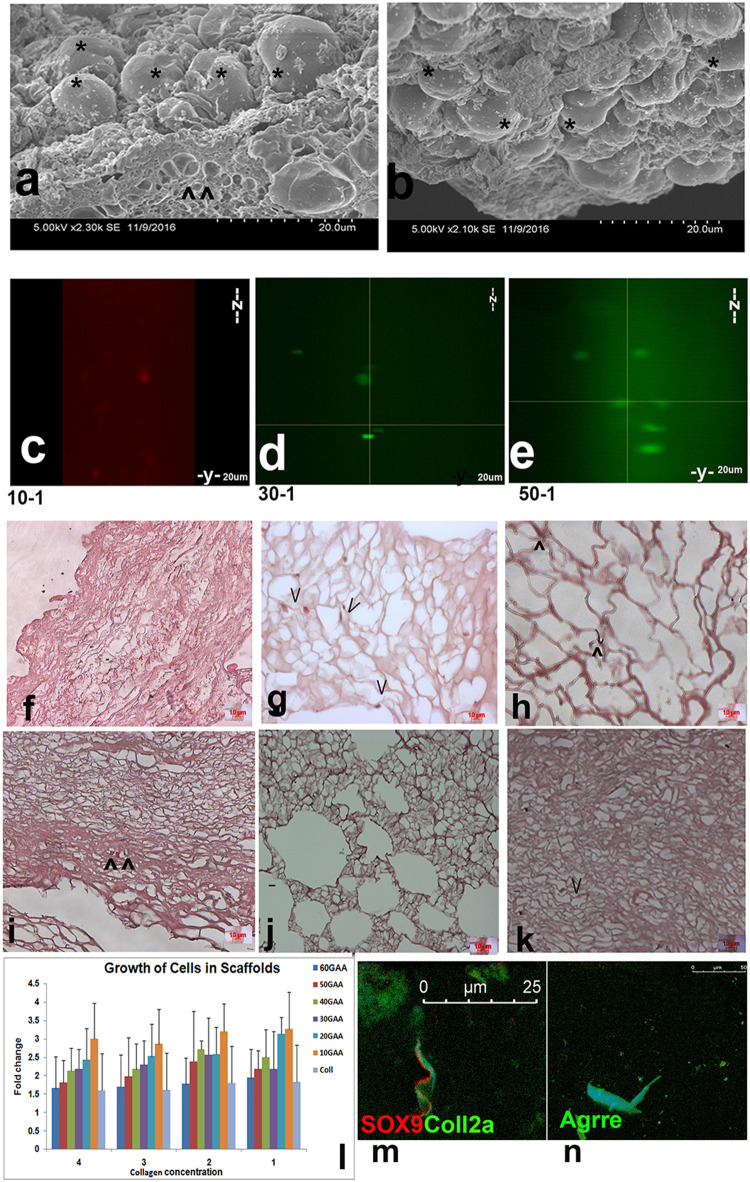
Scaffold cell interaction. **a**, **b** Scanning electron microscopy of gels incubated with human MSC show adherence of cells asterisk at the surface of the gels. **c**–**e** Cells were seen to penetrate inside the gels as seen in the confocal microscopy in the X–Z and Y–Z sections scans. The cells were preloaded with live tracker dyes and incubated with gels for 15 days. **f**–**k** Histopathology of scaffold blocks incubated for 30 days also shows presence of cells (^) in the section stained with hemotoxylin eosin, at higher oxidation levels the pores become narrow and cells are few. **l** The histogram depicting percent growth of cells in various GACO gels over a period of 30 days. At higher oxidation levels with higher concentration of collagen the cells growth seems to be lower. **m**–**n** NP Cells obtained from inter-vertebral discs also show survival and lineage retention in GACO gels. Cells stained positive for (**m**) sox9 and collagen 2a and (**n**) aggrecan

Cells are the main components in tissue engineering by their survival and lineage maintenance in a natural or synthetic scaffold. GACO scaffold series prepared with different concentrations of collagen were incubated with mesenchymal stem cells. Adherence of the cells to the scaffolds and their viability over a period of time was tested. The GACO blocks were washed with ethanol and UV-sterilized. Blocks were rehydrated and MSC derived from placenta were added to the dish. The cells were adhered to the scaffold not only on the surface, one could show the cells deep into the scaffolds (Fig. 6). Scanning electron microscopy studies showed the cells adhered to the blocks of scaffold. The cells could be seen blocking the pores as blobs on the scaffold surface (Fig. 6a, b). Scanning electron microscopy studies showed the cells adhered to the blocks of scaffold. The cells could be seen blocking the pores as blobs on the scaffold surface (Fig. 6a, b). Cells preloaded with tracking dyes were used to locate cells across the depth of scaffolds using confocal microscope; imaging showed the presence of cells at various depth levels in z plane (Fig. 6c–e). The histopathology studies also showed the presence of cells in different heights suggesting good penetration of cells in a highly porous scaffold (Fig. 6f–k). Cells not only adhered on the surface but could be seen penetrating the scaffolds and spread along the thin walls of the pores deep inside the scaffolds. Cells were not freely seen in the pores but adhering to the walls. Penetration of the cells inside the gels was slightly affected at higher collagen concentration of 4%.

MTT assay revealed the viability of cells in the scaffolds based on the metabolic activity in the cells. The number of cells attached to the scaffolds was as good as cells adhering to the culture dish. The proliferation of cells by 4 days was not very high but the cells remained viable (Fig. 6). The scaffold structure was biocompatible as the cells remained viable upon longer incubations toward 30 days. The MTT assay of the scaffolds incubated with cells for over 30 days revealed the presence of viable cells. Difference in the viability of cells at different combinations of gel was expressed as percent growth in each scaffold. With increased cross-linking, the proliferation of the cells decreased at each concentration of collagen with least growth in the gels cross-linked with 60% GAA. At higher cross-linking, one finds lesser numbers of cells penetrating the scaffold and lesser proliferation. Even with increase in collagen concentration, the penetration of cells was reduced. This was also evident in the histology as one could hardly locate cells in gels cross-linked with GAA at higher oxidation levels. All the constructs were biocompatible as the cells not only remained viable but also showed proliferation upon longer incubations for 30 days. This clearly demonstrated that the scaffolds were quite non-cytotoxic, biocompatible and bioconducive.

Seeding the lineage- and tissue-specific progenitors, derived from patient’s normal tissue or donor, into scaffolds is a rapidly expanding tissue engineering alternative as cells enclosed in a scaffold ensure a longer stay or implantation at the injury site. These gels were checked for their biocompatibility for nucleus pulposes (NP) cells obtained from surgical samples after obtaining approval. These cells in vivo survive in matrix that have a high glycan to protein ratio of about 27:1 (Mwale et al. 2004). Our results were quite encouraging as the cells remained alive and retained the markers of NP cells after 30 days in culture (Fig. 6m, n). The cells showed positive staining for Sox9, collagen 2a and aggrecan function which are the markers for NP cells.

Extracellular matrix is a composite of the proteins and the proteoglycans secreted locally by cells, maintaining homeostasis by virtue of endurance, and directing cell phenotypes and physiology. Using components of the ECM to create a scaffold was always preferred to avoid immunogenicity and toxicity. We have combined collagen, a major ECM protein with a plant-derived exudate to create a stable biocompatible scaffold. The composition of the gel can be varied by changing the oxidation level of the cross-linking polysaccharides that also alter the stability of the gels and can be useful in creating scaffolds according to the need of the cell types. In fact, one could also include other matrix proteins like laminin or fibronectin, etc. and custom-design the scaffolds. These mechanically stable gels could then be used in various therapeutic applications of degenerative diseases.

## Conclusion

Stable collagen scaffolds can be prepared by cross-linking with the oxidized gum arabic due to formation of covalent cross-links. Changes in the level of oxidation of gum arabic can be utilized as a tool to alter the composition of scaffold gels to suit the composition of ECM as required by the cells. The gels showed good stability, porosity, swelling capacity and high polysaccharide incorporation depending on the level of oxidation of gum arabic. These gels across the series were biocompatible and bioconducive though at high collagen concentration of 4% both cell penetration and growth was less. The process can be used to match cell/tissue ECM by mixing variety of tissue-specific ECM proteins/growth peptides and stabilize the composition with oxidized carbohydrate crosslinking; the gels could be designed according to the ECM requirement of tissues and used for tissue engineering applications.
